# Immunization with Transgenic Rodent Malaria Parasites Expressing Pfs25 Induces Potent Transmission-Blocking Activity

**DOI:** 10.1038/s41598-017-18831-8

**Published:** 2018-01-25

**Authors:** K. A. Sala, F. Angrisano, D. F. Da, I. J. Taylor, T. S. Churcher, A. M. Blagborough

**Affiliations:** 1 Department of Life Sciences, Sir Alexander Fleming Building, Imperial College London, Imperial College Road, South Kensington, London, SW7 2AZ UK; 2 Institut de Recherche en Sciences de la Santé, 399 Avenue de la Liberté, BP 545 Bobo-Dioulasso, Burkina Faso; 3 Jenner Institute, The University of Oxford, Roosevelt Road, Oxford, OX9 2PP UK; 4 MRC Centre for Outbreak Analysis and Modelling, Department of Infectious Disease Epidemiology, Imperial College London, Norfolk Place, London, W2 1PG UK

## Abstract

An anti-malarial transmission blocking vaccine (TBV) would be an important tool for disease control or elimination, though current candidates have failed to induce high efficacy in clinical studies. The ookinete surface protein P25 is a primary target for TBV development, but heterologous expression of P25 with appropriate conformation is problematic and a pre-requisite for achieving functional titers. A potential alternative to recombinant/sub-unit vaccine is immunization with a non-pathogenic, whole-parasite vaccine. This study examines the ability of a purified transgenic rodent-malaria parasite (*Pb*Pfs25DR3), expressing *Plasmodium falciparum* P25 in native conformation on the *P. berghei* ookinete surface, to act as a TBV. Vaccination with purified *Pb*Pfs25DR3 ookinetes produces a potent anti-Pfs25 response and high transmission-blocking efficacy in the laboratory, findings that are then translated to experimentation on natural field isolates of *P. falciparum* from infected individuals in Burkina Faso. Efficacy is demonstrated in the lab and the field (up to 93.3%/97.1% reductions in transmission intensity respectively), with both a homologous strategy with one and two boosts, and as part of a prime-boost regime, providing support for the future development of a whole-parasite TBV.

## Introduction

To eradicate malaria, we must reduce disease within individuals, and inhibit transmission of *Plasmodium* through populations^1^. The dynamics of the malaria life-cycle indicate that transmission reduction will be most effective when targeting the parasite within the mosquito^2,3^. Anti-malarial transmission-blocking vaccines (TBVs) have shown promise as an effective means to reduce transmission. In this strategy, antibodies are ingested by the mosquito during a bloodmeal and prevent parasite development by binding to the surface proteins of gametocytes, gametes, zygotes/ookinetes^4,5^, or to mosquito-derived antigens expressed within the mosquito midgut (e.g. FREP1^6^). A primary target for TBV development is the P25 protein, expressed on the surface of the zygote/ookinete^7^. The P25 family of proteins is characterized by the presence of EGF-like motifs, multiple cysteines, and a complex tertiary structure^8^, making them challenging to express with accurate native conformation. These proteins, whilst GPI anchored, are otherwise un-glycosylated in *Plasmodium*^9^, whereas eukaryotic expression systems typically glycosylate P25. Phase I trials of TBVs against *Plasmodium falciparum* P25 (Pfs25) have been performed previously^10,11^ and field trials are underway, with modest efficacy reported so far. This is widely hypothesised to be due to lack of correctly folded antigen with “correct” conformation/post-translational modification, and appropriate immunogenicity^10–13^.

To develop an effective TBV successfully, choosing a target antigen is only the first step. The need to express protein with native conformation and find a potent production/delivery system, complemented by appropriate formulation, adjuvant, dosages and immunization regimes is also vital. Various methods for P25 expression have been used in the past, including *E. coli*, *L. lactis*, Baculovirus, yeast, DNA administration and algae^12–19^. Delivery technologies such as virus-like/nano-particles have also been used^20–23^. Despite this, expression of recombinant P25, with appropriate glycosylation, conformation and antigenic presentation is problematic, and a potential pre-requisite for achieving high functional titers in humans. This is a clear technical bottleneck for successful vaccine development^9,23–25^.

An alternative to heterologous expression of recombinant/sub-unit based antigens is the use of whole-parasite vaccines, which naturally exhibit native expression of antigens. Typically, these vaccines use radiation or chemically-attenuated/killed parasites to induce an effective immune response. Although successfully demonstrated on a small scale in the 70 s^26–29^, this approach was initially deemed impractical and focus shifted to recombinant antigens. However, the success of researchers in recent years to manufacture/purify parasites to clinical grade and use them to develop vaccines that show protection in humans against infection has caused a re-evaluation^30–35^, generating clinical-grade, purified, irradiated or chemoattenuated sporozoites to act as potential pre-erythrocyte vaccines (PfSPZ). Trials of PfSPZ have demonstrated that whole-parasite vaccines, produced to GMP quality, can be well tolerated, immunogenic, and result in significant protection from infection in the lab^36^ and the field^37^. An alternative approach to induce *P. falciparum* sporozoite immunity is ongoing, using a transgenic rodent malaria lines (e.g. PbPfCS@UIS4^38,39^). Whole-parasite vaccine approaches are also being extensively explored against the blood stages of the lifecycle, using chemically attenuated^40^, genetically attenuated^41^, irradiated^42^ or killed^43^ parasites.

Comparatively, efforts to generate a whole-parasite TBV have lagged. Previously, we have developed a transgenic rodent-malaria (*Plasmodium berghei*) line, *Pb*Pfs25DR3, expressing (human-malaria) *P. falciparum* Pfs25 in place of its rodent homologue (Pbs25). *Pb*Pfs25DR3 is phenotypically indistinguishable to WT *P. berghei*, expresses Pfs25 on the surface of the zygote/ookinete, and has been used previously to assay a range of TBVs^16,17^. Crucially, in this parasite, Pfs25 is expressed at high levels in native conformation, as demonstrated by functional complementation of its rodent homologue and the use of conformation dependent antibodies to assess expression^16,17^. The use of “humanized” rodent malaria parasites is recognized as a safe, cost-effective, and simple method to investigate the potency of anti-malarial vaccines^16–19^, however, their ability to induce a transmission-blocking response as part of a whole-organism vaccine has not been assessed. Immunization of these parasites will not act as a “traditional” anti-malarial whole parasite vaccine - ookinetes cannot establish infection in the vertebrate host. As a result, immunity due to the exploitation of natural infection of the host will not occur as with attenuated sporozoite or merozoite whole-parasite vaccines^40–43^. Conversely, the presentation of functionally competent Pfs25 antigen with proven native conformation is logically advantageous in terms of generation of a functional anti-parasitic immune response, and can potentially address concerns regarding the heterologous expression of malaria vaccine candidate antigens^9,24,25^.

Here, we have performed experiments to assess the ability of immunization with *Pb*Pfs25DR3 to initiate a transmission blocking response. A range of clinical adjuvants, doses and immunization regimes are examined; Alhydrogel, a common aluminium hydroxide wet-gel suspension is used as adjuvant, as is Matrix M, an adjuvant containing 40 nm nanoparticles composed of Quillaja saponins/cholesterol/phospholipid. Matrix M has previously demonstrated utility when used with Pfs25-based vaccine candidates^23^. Additionally, the ability of *Pb*Pfs25DR3 to act as “boost” within a “prime-boost” strategy was assessed, using a lead Pfs25 adenoviral vaccine (ChAd63-Pfs25) as the “prime” dose. Viral vectors have been shown to reliably induce functional antibodies against a range of malaria antigens in both pre-clinical and clinical studies in addition to being a reproducible means to induce cellular immunity. ChAd63 (chimpanzee adenovirus 63) has been used to express Pfs25 (and other TBVs previously), and when administered in a heterologous prime-boost regime, has elicited anti- parasitic moieties that are capable of blocking transmission in membrane-feeding assays^16,17^. Using these systems, we demonstrate that immunization with purified *Pb*Pfs25DR3 ookinetes elicits a potent anti-Pfs25 immune response, inducing antibodies that recognize native protein on the ookinete surface. We also demonstrate that *Pb*Pfs25DR3 immunization can initiate an anti-malarial transmission-blocking response with high efficacy, both *in vivo* in immunized mice, and by direct membrane feeding assay (DMFA), *ex vivo* at a range of infection densities, on blood samples from infected African blood donors. This data provides support for the future potential development of a whole-parasite TBV.

## Results

### Immunization with PbPfs25DR3 results in induction of anti-Pfs25 antibodies which recognize antigen in native conformation

**Figure 1:**
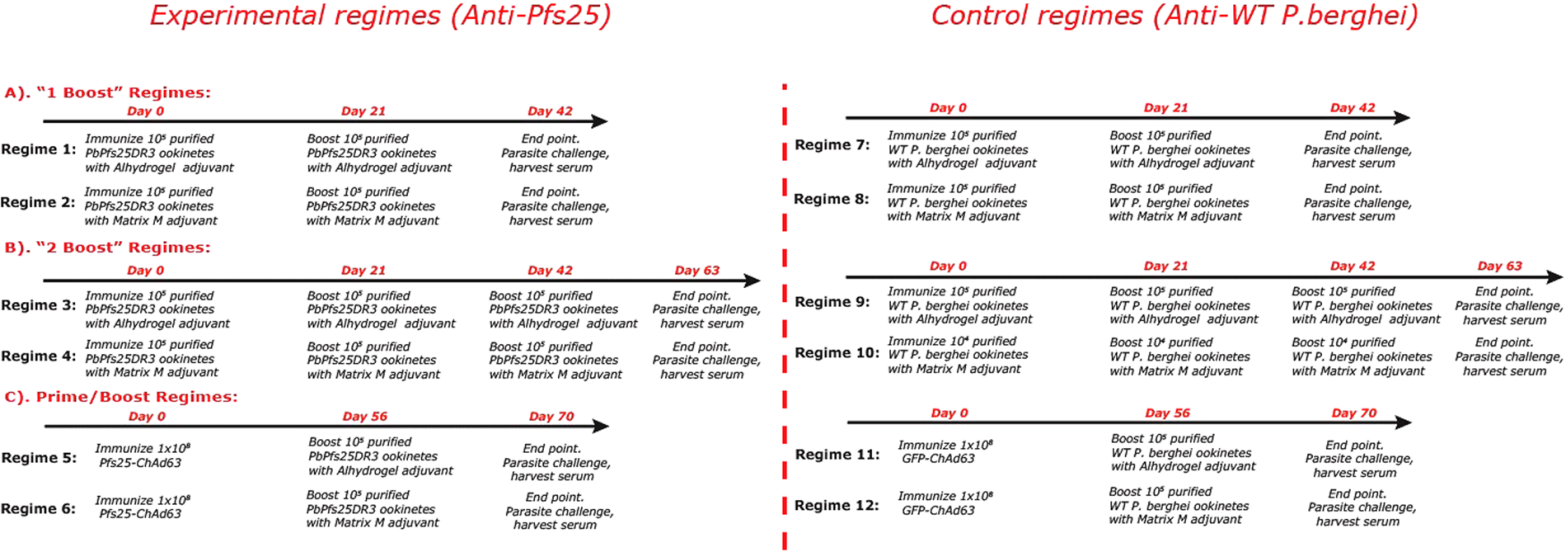
Anti-*Pb*Pfs25DR3 immunization regimes. Groups of 5 mice received each vaccine regime. For each individual experimental regime, the corresponding (negative) control regime is to its immediate right. In each regime, for DFA 5 mice were challenged with *P. berghei Pb*Pfs25DR3 to assess for transmission blockade. In regimes 1–6 mice were immunized to attempt to induce a Pfs25 response (‘experimental regimes’). In regimes 7–12 mice were immunized with carrier protein or empty vector controls (‘control regimes’). Regimes 1,2,7,8 use Matrix M as adjuvant, 3,4,9,10 use alhydrogel, 5,6,11,12 are prime/boost regimes, with ChAd63 prime and ookinete boost. All immunizations were performed *i.m*.

In all regimes (Fig. 1), immunization with *Pb*Pfs25DR3 ookinetes results in induction of antibodies that recognize Pfs25 to varying degrees. To examine immunogenicity, end-point sera were examined by ELISA against recombinant (*Pichia* expressed) r-Pfs25. Sera were taken from individual mice (n = 5) and examined for anti-Pfs25 responses. Responses against r-Pfs25 were not detected when examining non-immunized serum. Immunization with purified WT ookinetes in all control regimes (7–8) resulted in no visible induction of anti-Pfs25 antibodies with any adjuvant/delivery systems tested here, suggesting a lack of cross-species induction of anti-P25 antibodies (against Pbs25 or Pbs28).

**Figure 2:**
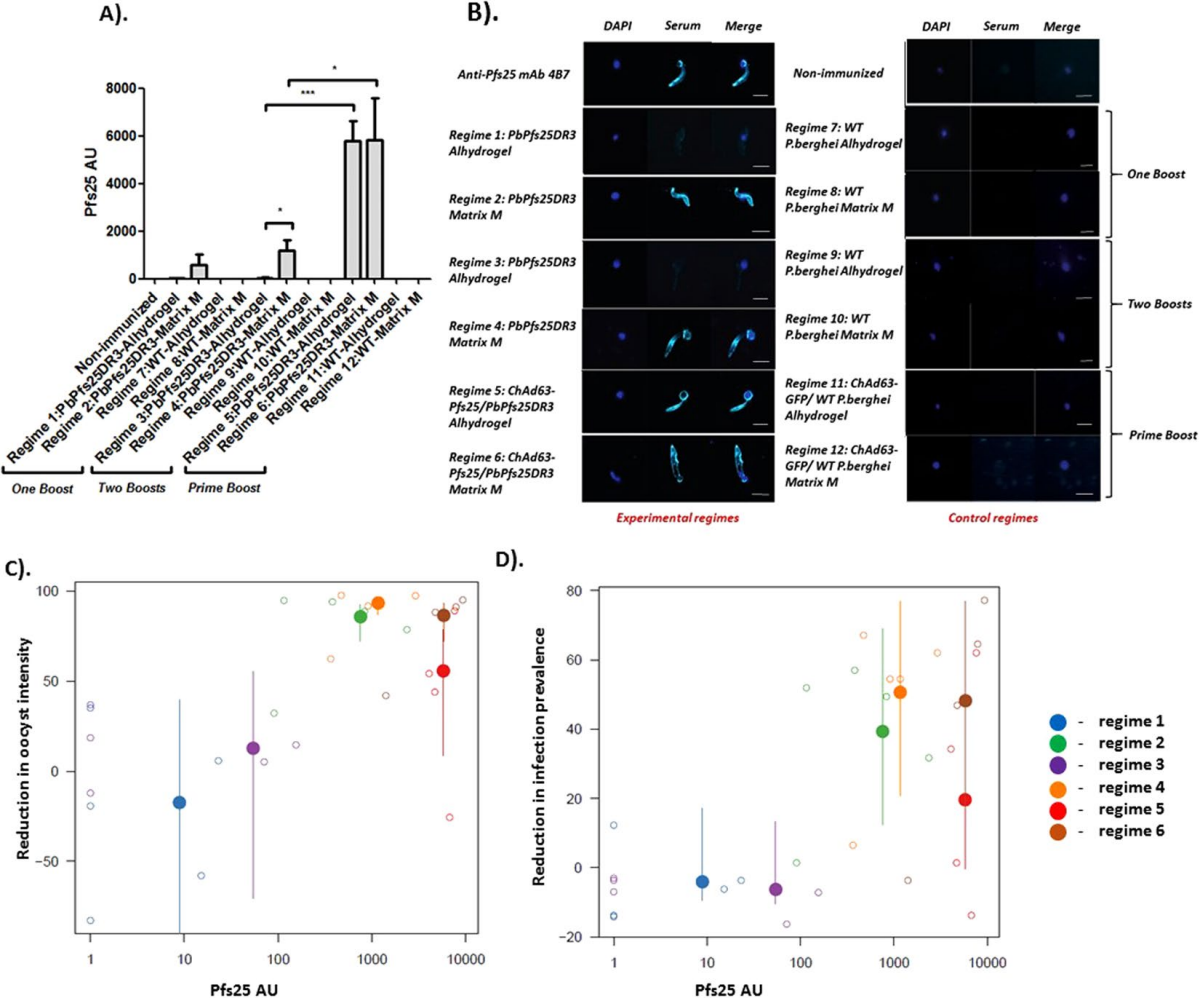
Induction of antibody following immunization with *Pb*Pfs25DR3 ookinetes. The ability of each regime to generate Pfs25-specific antibody responses after administration was tested by ELISA against recombinant Pfs25 protein^12,17^ and IFA against *P. falciparum* (NF54) ookinete/retort stages within the mosquito midgut 26 hours post-feed. (**A**) *End -point anti-Pfs25 titers in serum*. Bars show mean titers from 5 mice. Pre-immune/non-immunized serum did not recognize recombinant Pfs25. Error bars represent SEM. Bars show mean titers from 5 mice. Error bars represent SEM. (**B**) *IFA against P. falciparum (NF54) ookinete/retort stages*. Ability of generated serum to recognize native Pfs25 on the surface of sexual stages of *P. falciparum* post-fertilization was assessed by immunofluorescence on fixed, non-permeabilized parasites probed with anti-serum from each regime. To control for non Pfs25-specific signal, IFA was performed using serum from control regimes (7–12). Each panel shows an overlay of anti-Pfs25 signal (turquoise) and DNA labelled with DAPI (blue). Scalebar  =  5 µm. (**C** and **D**) *The ability of induced titers to reduce both oocyst intensity (C) and infection prevalence (D) by DFA, and relationship with anti-Pfs25 titers*. Small open circles denote estimates generated from mosquitoes fed on individual mice (Fig. 3) compared to the average from mosquitoes feeding on unimmunized mice) whilst the large filled dots show the average for the regimen as estimated using a generalized-linear mixed effect model. Vertical lines show 95% confidence interval on overall estimates. Point colours indicate the regimen tested, be it regime 1 (blue), 2 (green), 3 (purple), 4 (orange), 5 (red) or 6 (brown).

The lowest anti-Pfs25 antibody titers were observed when Alhydrogel was used as adjuvant (regimes 1 and 3) and higher titers were observed with Matrix M (regimes 2 and 4). When one boost was performed (1 and 2), no significant difference was observed in titer if either Alhydrogel or Matrix M was used. Conversely, if two boosts were performed (3 and 4), significantly higher titers were achieved with Matrix M (*p* = 0.0417) (Fig. 2A). For all regimes immunizing with ookinetes alone (in the absence of adenovirus), no significant difference in titer was observed between 1 and 2 boosts. Anti-Pfs25 responses rise dramatically when an adeno-prime/ookinete boost strategy is utilized. ChAd63-Pfs25-prime, *Pb*Pfs25DR3/Alhydrogel boost results in a significantly higher mean titer than the *Pb*Pfs25DR3/Alhydrogel 2 boost strategy (P =  < 0.0001). A significant difference in titers is also observed when comparing *Pb*Pfs25DR3/Matrix M-2 boost and ChAd63-Pfs25-prime, *Pb*Pfs25DR3/Matrix M boost (*p* = 0.0241). Titers are equivalent to those previously observed with immunization with ChAd63-MVA-Pfs25^17^, but less than those observed with ChAd63-MVA-Pfs25-IMX313^23^. When a prime-boost strategy is performed, no significant difference in anti-Pfs25 titer is observed between Alhydrogel and Matrix M (regimes 5 and 6).

To confirm the induction of anti-Pfs25 antibodies that recognize native protein on the surface of the parasite, pooled end-point serum was used to perform IFA on mosquito-derived *P. falciparum* ookinetes/retorts. Parasite-specific surface staining corresponding to the established expression of Pfs25, and identical to that observed with anti-Pfs25 mAb 4B7, was observed using sera from regimes 2, 4, 5 and 6 (Fig. 2B), containing Matrix M as adjuvant. Conversely, only very low intensity signal was observed with regimes utilizing Alhydrogel. Control immunizations (7–12) resulted in no observable fluorescence.

### Immunization with PbPfs25DR3 results in a potent transmission-blocking effect *in vivo*

**Figure 3:**
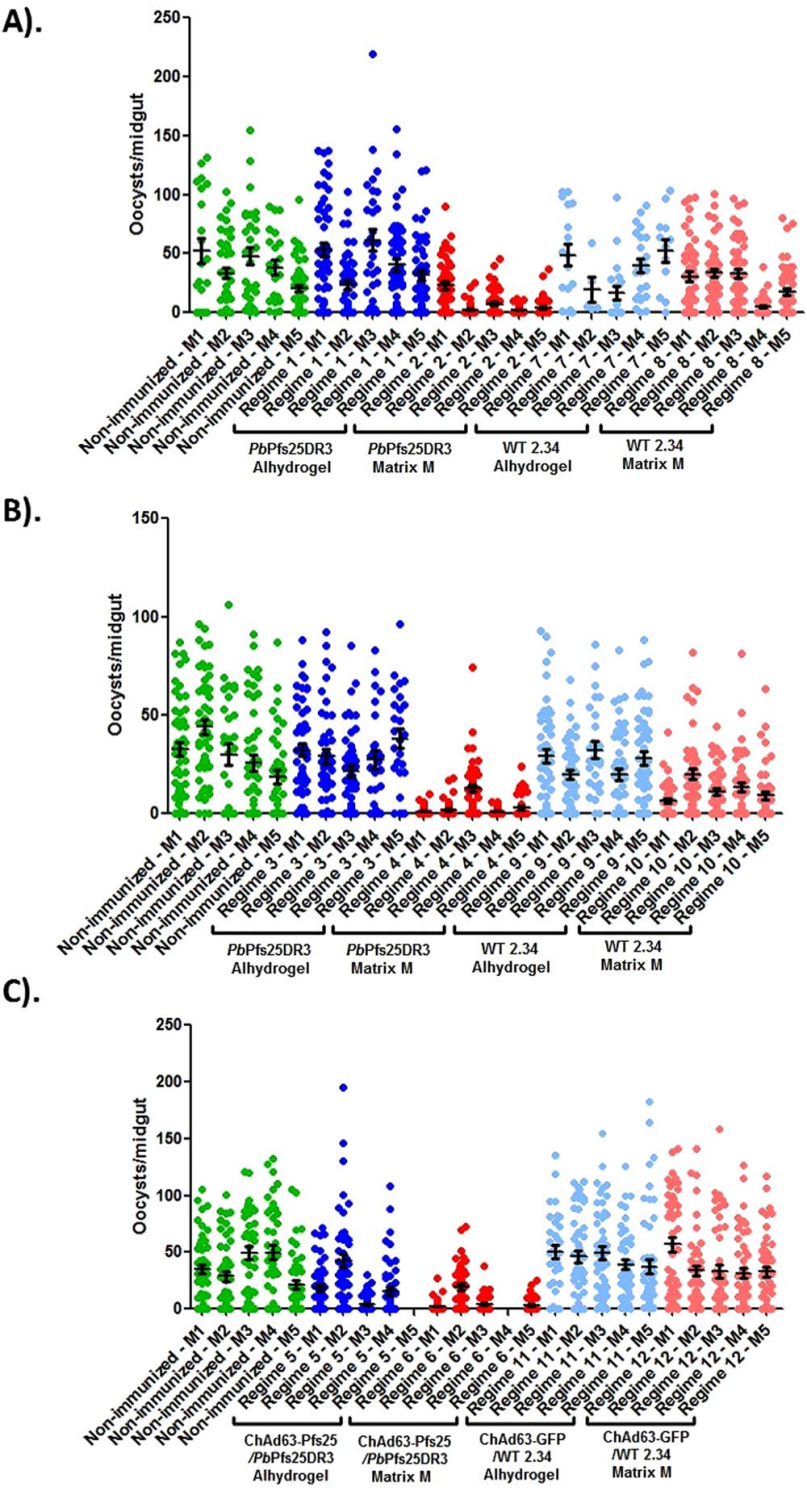
Assessment of *in vivo* transmission blockade following immunization with *Pb*Pfs25DR3 ookinetes by DFA. To assess transmission-blocking activity following immunization, 5 mice per regime (experimental and control) were infected/challenged with *P. berghei* PbPfs25DR3 and three days later, mosquitoes were exposed to individual mice to perform DFA. DFAs were performed in three individual tranches to account for varying experimental timings within multiple regimes (**A**) regimes 1,2,7,8; (**B**) regimes 3,4,9,10; (**C**) regimes 5,6,11,12). Transmission blockade was assessed as mean reduction in oocyst intensity/ prevalence with respect to cohorts of non-immunized challenged mice within each DFA. Regimes 5 and 6 contain four mice due to death before challenge. Individual data points represent the number of oocysts found in individual mosquitoes 12 days post feed. Horizontal bars indicate mean intensity, whilst error bars indicate S.E.M. For each regime, mean reductions in intensity and prevalence for each group are reported in Table 1.

To examine induced transmission blocking efficacy following immunization, 5 mice per regime were mechanically infected with *P. berghei Pb*Pfs25DR3, following which, direct feeding on individual mice was performed (Fig. 3). Adverse effects on rodent health were not observed following immunization with any regime. Specifically, pallor, piloerection, reduced mobility, lethargy/weakness, weight loss compared to cage mates, or respiratory distress was not observed in any immunized mice. Mean reductions in intensity and prevalence are reported in Table 1. In the control regimes (regimes 7–12), no significant transmission blocking efficacy was observed. The exception to this was observed with regime 10 (WT 2.34 Matrix M – 2 boosts), potentially due to the induction of non-Pfs25, uncharacterized *P. berghei* ookinete-specific transmission-blocking moieties under these conditions.

**Table 1:** Overall *in vivo* transmission-blockade following immunization with *Pb*Pfs25DR3 ookinetes by DFA.

Regime	Total mice	Total mosquitoes	Mean oocyst intensity (±SEM)	Mean infection prevalence	Mean reduction in intensity	Mean reduction in prevalence
**1:** *Pb*Pfs25DR3 Alhydrogel – 1 boost	5	250	41.96 (5.6)	83.2%	−17.4%	−4.1%
**2:** *Pb*Pfs25DR3 Matrix M – 1 boost	5	250	7.56 (1.1)	48.4%	85.7%***	39.3%***
**3:** *Pb*Pfs25DR3 Alhydrogel – 2 boost	5	199	29.53 (3.8)	85%	12.8%	−6.3%
**4:** *Pb*Pfs25DR3 Matrix M – 2 boost	5	250	3.82 (0.7)	50%	93.3%***	50.6%***
**5:** *ChAd63-Pfs25/Pb*Pfs25DR3 Alhydrogel	4	200	20.14 (3.3)	62.5%	55.7%***	19.6%***
**6:** *ChAd63-Pfs25/Pb*Pfs25DR3 Matrix M	4	200	7.1 (1.3)	42.5%	86.5%***	48.2%**
**7:** WT 2.34 Alhydrogel – 1 boost	5	77	35.41 (8.3)	87.6%	−5.3%	−5.4%
**8:** WT 2.34 Matrix M – 1 boost	5	250	24 (3.1)	79.2%	38.9%	−0.2%
**9:** WT 2.34 Alhydrogel – 2 boost	5	227	25.8 (3.3)	80.1%	24.1%	−0.2%
**10:** WT 2.34 Matrix M – 2 boost	5	250	12.01 (1.9)	75.6%	66.1%*	4.2%
**11:** *ChAd63-GFP/* WT 2.34 Alhydrogel	5	230	44.5 (5.5)	91.6%	−33.2%	−14.9%
**12:** *ChAd63-GFP/* WT 2.34 Matrix M	5	250	37.6 (5.1)	83.6%	−10.6%	−5.2%

Immunization with *Pb*Pfs25DR3/Alhydrogel results in no significant efficacy in terms of reduction in intensity or prevalence. Conversely, immunization with *Pb*Pfs25DR3/Matrix M preparations results in significant and potent levels of blockade, with an 85.7% reduction in intensity/39.3% reduction in prevalence observed with a single boost (regime 2). When the number of boosts is increased, reduction in intensity/prevalence of 93.3% and 50.6% respectively are achieved (regime 4). No significant difference between performing one boost or two boosts was observed when immunizing with *Pb*Pfs25DR3/Matrix M, in terms of inhibition of intensity (*p* = 0.072) or prevalence (*p* = 0.431). When immunizing *Pb*Pfs25DR3 with Alhydrogel or Matrix M as adjuvant as part of a “prime-boost” regime, significant efficacy is also observed. For regime 5 (ChAd63-Pfs25-prime/*Pb*Pfs25DR3 Alhydrogel-boost), reductions in intensity/prevalence of 55.7%/19.6% were achieved. This efficacy was considerably higher when using ChAd63-Pfs25-prime/*Pb*Pfs25DR3 Matrix M-boost (regime 6), with an 86.5%/48.2% reduction in intensity/prevalence observed. Significant differences between regime 2 (*Pb*Pfs25DR3/Matrix M 1 boost) or regime 4 (*Pb*Pfs25DR3/Matrix M 2 boosts) and regime 6 (ChAd63-Pfs25-prime/*Pb*Pfs25DR3 Matrix M-boost) were not observed, in terms of inhibition in intensity or prevalence. When considering blockade observed in individual immunized mice, transmission blocking efficacy is linked to induced anti-Pfs25 titer (Fig. 2C,D), with a significant relationship between reduction in intensity (*p* = 8.99 × 10^−8^) and prevalence (*p* = 2.56 × 10^−6^) and titer.

### Serum derived from immunization with PbPfs25DR3 results in transmission-blocking efficacy against field isolates of P. falciparum

**Figure 4:**
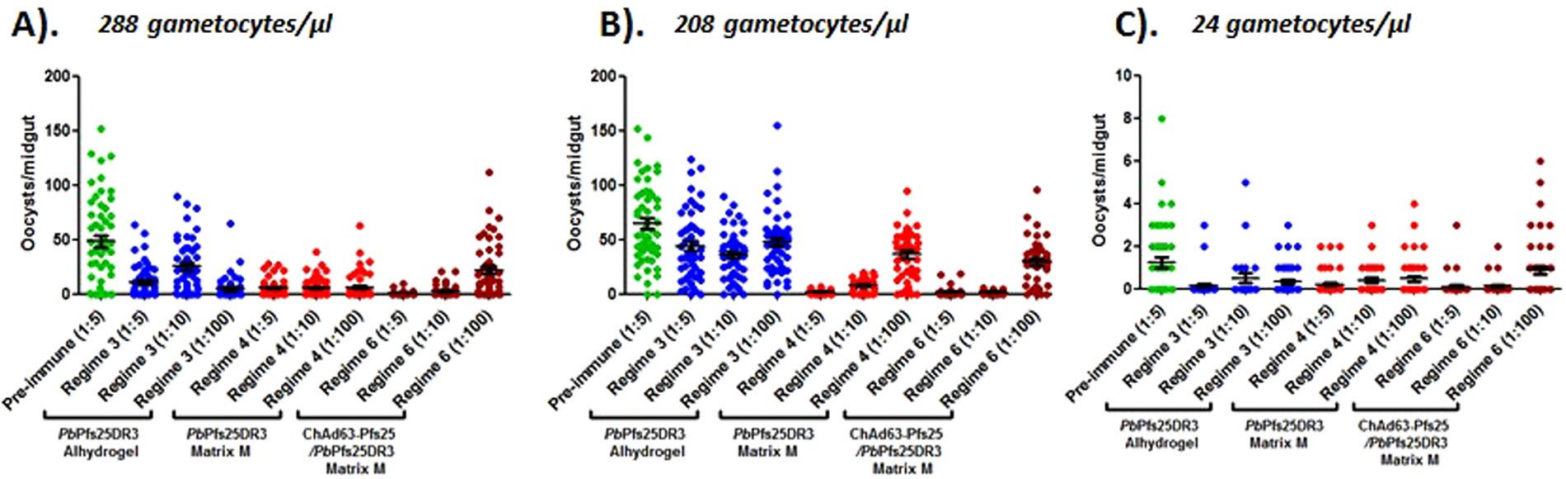
Transmission blocking efficacy of serum derived from immunization with *Pb*Pfs25DR3 against field isolates of *P. falciparum*. To assess the ability of serum generated from regimes 3 (*Pb*Pfs25DR3/Alhydrogel 2 boosts), 4 (*Pb*Pfs25DR3/Matrix M 2 boosts) and 6 (ChAd63-Pfs25-prime/*Pb*Pfs25DR3 Matrix M-boost) to block transmission of malarial field isolates, *P. falciparum* gametocytes were collected from naturally infected volunteers recruited in malaria endemic localities, and DMFA subsequently performed. A range of naturally occurring gametocyte densities were assessed (**A**,**B**,**C**) were examined at dilutions of 1:5, 1:10 and 1:100, and control (pre-immune) serum at 1:5. Individual data points represent the number of oocysts found in individual mosquitoes 12 days post feed. Horizontal bars indicate mean intensity of infection, whilst error bars indicate S.E.M. within individual samples. For each regime, the mean reductions in intensity and prevalence at each dilution are reported in Table 2.

To assess ability to block transmission of field isolates, *P. falciparum* gametocytes were collected from naturally infected volunteers, and DMFA performed (Fig. 4, Table 2). Multiple gametocyte densities were assessed. Serum from regimes 3, 4 and 6 were examined at dilutions of 1:5, 1:10 and 1:100.

**Table 2:** Overall transmission-blocking efficacy of serum derived from immunization with *Pb*Pfs25DR3 against field isolates of *P. falciparum*.

	PbPfs25DR3 Alhydrogel – 2 boost			PbPfs25DR3 Matrix M – 2 boost			ChAd63-Pfs25/PbPfs25DR3 Matrix M		
	1 in 5	1 in 10	1 in 100	1 in 5	1 in 10	1 in 100	1 in 5	1 in 10	1 in 100
***Expt. A:***									
Inhibition in intensity (%)	77.3%***	47.8%**	90.3%***	89.1%***	87.7%***	87.3%***	98.3%***	94.5%***	54.8%**
Inhibition in prevalence (%)	9.5%	0%	38.1%***	19.1%	26.2%	42.0%*	59.5%***	40.5%**	4.7%
***Expt. B:***									
Inhibition in intensity (%)	32.5%**	45.6%***	27.0%*	96.8%***	87.3%***	64.7%***	97.3%***	96.1%***	46.6%***
Inhibition in prevalence (%)	0%	2.0%	−1.9%	22.0% *	13.7%	4.7%	46.0%***	26.1%**	5.9%
***Expt. C:***									
Inhibition in intensity (%)	89.0%***	59.4%**	71.7%*	83.1%***	68.5%*	62.3%*	91.5%***	90.8%***	29.7%
Inhibition in prevalence (%)	89.1%***	50%**	52.1%*	70.8%**	41.9%	47.6%*	87.5%***	83.3%***	7.7%
***Overall inhibition:***									
Inhibition in intensity (%)	62.5%**	46.9%*	65.2%***	90.7%***	85.6%***	69.1%***	97.7%***	94.7%***	46.6%***
Inhibition in prevalence (%)	22.7%*	9.3%	32.0%***	35.6%***	28.9%**	32.7%***	71.2%***	51.0%***	5.48%

Intensity and prevalence were profoundly reduced by addition of immune serum, in comparison with feeds supplemented with pre-immune (control) serum. The highest reductions in intensity/prevalence were observed with regime 6, where a 97.7%/71.2% reduction was observed at a serum dilution of 1:5. Significant inhibition was demonstrated at lower dilutions. Feeds supplemented with serum from regime 4 also resulted in high levels of inhibition (90.7%/35.6%) at a dilution of 1:5, again, with significant inhibition demonstrated at lower dilutions. Across all dilutions, no significant difference between reduction in intensity or prevalence was observed when comparing regimes 4 or 6. Lower, but significant, levels of inhibition were seen using serum from regime 3 at dilutions of 1:5 and 1:100. Efficacy from regime 3 was lower than that observed with regimes 4 and 6, but higher than that observed with *in vivo* immunization/challenge in the DFA (Fig. 3). This is potentially due to higher than physiological concentrations of anti-Pfs25 moieties or reduced parasitic densities in the *ex vivo* DMFA, compared to the *in vivo* DFA.

## Discussion

This study describes the establishment and assessment of a novel whole-parasite transmission blocking vaccine, using a transgenic rodent malaria parasite, *Pb*Pfs25DR3^16^ expressing the human malaria antigen Pfs25, on the surface of the ookinete. *Pb*Pfs25DR3 expresses biologically competent Pfs25 with native conformation, evidenced by its ability to enable transmission in the absence of Pbs25 and Pbs28 (both P25 and P28 are mutually redundant, and loss of both proteins without appropriate complementation results in absence of transmission *ex vivo* and *in vitro*^44^). We demonstrate that following immunization with *Pb*Pfs25DR3, anti-Pfs25 responses are initiated, and that induced antibodies have Pfs25-approriate conformation, evidenced by their ability to recognize the ookinete/retort surface of *P. falciparum*. We show that immunization with purified *Pb*Pfs25DR3 ookinetes confers significant transmission-blocking immunity, assessed directly *in vivo*, and *ex vivo*, on field isolates of *P. falciparum*. As described in previous studies, transmission-blocking efficacy is broadly related to anti-Pfs25 titer^45^.

To assess the induction of transmission-blocking responses, we utilized a range of regimes, doses and adjuvants. Two clinically relevant adjuvants were assessed, Alhydrogel, and Matrix M. Additionally, the ability of *Pb*Pfs25DR3 to act as the “boost” for a “prime-boost” strategy was assessed, using ChAd63-Pfs25 as “prime”. Our DFA results indicate that regimes using Alhydrogel as adjuvant do not result in high efficacy, whereas Matrix M results in a potent effect, whether used with a single boost (regime 2), two boosts (regime 4) or as part of a prime-boost (regime 6). Maximal efficacy was achieved with regime 4, with a 93.3% reduction in intensity and 50.6% reduction in prevalence, but significant differences in efficacy between regimes 2, 4 and 6 were not detected. This study used inbred Balb/C mice for immunization. Whereas the inbred nature of this rodent model allows for a more direct, head-to-head comparison between immunizations with different antigenic preparations (variation due to outbred effects is reduced), this may impact on the predictive nature of these studies to human studies. Further studies examining effects in outbred models maybe advantageous. High efficacy was also seen by DMFA on field isolates of *P. falciparum* with Matrix M-derived serum. Within the DMFA, dose-responsive inhibition of transmission was observed with serum derived from regimes 4 and 6, but not with regime 5 (*Pb*Pfs25DR3 Alhydrogel – 2 boost).

The potential uses and applications of whole-parasite vaccines against malaria have been well discussed on multiple occasions^24,25,46^. In the case of *Pb*Pfs25DR3, there are numerous advantages to its potential development as a TBV. Pfs25 is expressed in this format in proven native conformation, resulting in induction of antibody that recognizes native protein on the surface of ookinetes. Given that *Plasmodium* lacks many common post-translational modifications, previous attempts to express Pfs25 in a heterologous system have led to potential issues with unwanted glycosylation, impacting on functional transmission blockade^9,47^. The ability of antigen to form appropriate disulphide bonds is also key. An as yet uncharacterized number of disulphide bonds are essential for the function of Pfs25 as an immunogen^9^. Using a transgenic rodent *Plasmodium* parasite to express Pfs25 circumvents these issues, while maintaining high titers and production of functional antibody.

There remain multiple technical and safety challenges relating to the use of whole-parasite vaccines in a clinical setting. A general concern is the possibility of underattenuation, or reversion to wild type^46^. The use of *Pb*Pfs25DR3 makes this impossible; firstly, the ookinete form of the parasite is mosquito exclusive, not transmitted, and is unable to establish/propagate bloodstage infection. Secondly, *Pb*Pfs25DR3 was constructed using double homologous recombination^16^, making spontaneous reversion impossible. Thirdly, even if reversion was to occur, *P. berghei* is not a human pathogen, and cannot establish bloodstage infection in humans, and finally, ookinetes are dead when immunized. The use of mouse-derived blood products in the manufacturing process is an issue when considering immunization with purified *P. berghei* parasites, potentially leading to alloimmunization. This must be thoroughly assessed prior to clinical trials. Here, ookinetes were prepared by CF11 purification to remove white blood cells, and lysis of erythrocytes (by NH_4_Cl), followed by magnetic purification to remove contaminants^48^. These methods lead to ookinete isolation with high purity (~99%)^49^. Previous MALDI-TOF analysis of these preparations show low levels of contamination^50–52^, typically by mouse haemoglobin-β^52^. It is unknown whether purification at this level is appropriate to avoid alloimmunization, however, if necessary, multiple purification methods could be combined, including density-gradients^48,52^, antibody-conjugation^49^ or enhanced chromatography^53^. Further examination is clearly required to minimize an inappropriate immune response, but murine-derived proteins (e.g. Zevalin, Bexxar) have been successfully utilized as therapeutics for decades. Production/purification of *P. berghei* ookinetes is cheap, robust, and routinely performed at HTP scale^54^, with yields of 12 × 10^6^/ml, and proven scalability^52^. At the dose examined within this study (10^5^ ookinetes immunized), a single ml of blood will provide ~120 individual immunizing doses, however, this scalability is only currently proven for experimental purposes. Clearly, further investigation would be needed to explore feasibility to scale production to support a vaccine pipeline, and to manufacture a viable vaccine to GMP standard. Experiments to assess the long-term persistence of functional antibody post-immunization would be desirable in the future. Generally, as described in multiple previous studies^36–43^, it is widely accepted that the manufacture of whole-parasite vaccines will require innovative approaches that are unconventional in “traditional” vaccinology^25^.

Within this study, to expand the repertoire of effective whole-parasite anti-malarial vaccines, we performed experiments using a safe, cheap, and easily cultured transgenic rodent-malaria parasite expressing a clinically-relevant TBV antigen (Pfs25) on its surface in native conformation. Vaccination produces an anti-Pfs25 immune response, and induces a potent response *in vivo* and *ex vivo*, in the lab and against field isolates of *P. falciparum*. High efficacy is demonstrated with a single boost, and without implementation of a prime-boost strategy. This data provides strong support for the further development and assessment of a whole-parasite TBV in the future.

## Materials and Methods

### General Parasite Maintenance

General maintenance of *P. berghei* ANKA 2.34 (WT) and *Pb*Pfs25DR3 parasites was carried out as described previously^49,55^. The generation, genotyping and phenotypic analysis of *Pb*Pfs25DR3, where endogenous *Pbs*25 (and *Pbs*28) was replaced with *Pfs*25, was previously described^16^.

### Ookinete culture and purification

*P. berghei* ANKA 2.34 or *Pb*Pfs25DR3 ookinete culture and purification was performed as described in^16^. Purification of ookinetes was performed by passing cultures through a CF11 (Whatman) column to remove white blood cells, and erythrocytes were lysed by NH_4_Cl lysis^16^. Ookinetes were then purified on a VarioMACS Magnetic Cell Separator^48,49^ to remove contaminating cell debris. Purified ookinete preparations were aliquoted into individual doses and frozen to kill live parasites and preserve until immunization.

### Immunization regimes

Sixty mice (Female BALB/c, 6–8 weeks of age (Harlan, UK)) were divided into twelve groups of 5 mice (Fig. 1). Groups 1–6 were experimental whereas groups 7–12 were immunized (*i.m*.) with control immunogens (either non-transgenic, WT 2.34 ookinetes to control for immunization with *Pb*Pfs25DR3 ookinetes, or ChAd63-GFP to control for immunization with ChAd63-Pfs25^17^. Transmission blockade observed following immunization with WT 2.34 ookinetes or ChAd63-GFP was non-specific, and not due to immunization with Pfs25.

For Alhydrogel vaccination, doses were prepared by combining 50 µL of Alhydrogel with 1 × 10^5^ purified ookinetes. For Matrix M (Novavax), 1 × 10^5^ ookinetes were mixed with adjuvant, vortexed and injected (12 µg Matrix M dose per mouse in total volume). Adenoviral vaccines were prepared in sterile, endotoxin-free PBS with doses of 1 × 10^8^ viral particles of ChAd63 vaccines at day 0. Endpoint serum was collected for ELISA at day 42 for regimes 1, 2, 7 and 8; at day 63 for regimes 3, 4, 9 and 10; and at day 70 for regimes 5, 6, 11 and 12 (adeno-prime, ookinete-boost).

### Enzyme-linked Immunosorbent Assay (ELISA)

Nunc-Immuno Maxisorp 96-well plates (NUNC) were coated with 100 ng recombinant protein per well (r-Pfs25, described in^12,17^). After blocking with 5% skimmed-milk, sera were incubated for 2 hours followed by goat anti-mouse IgG-AP (Sigma), 1:5000. Plates were developed using pNPP and read at OD_405_ until set end-point detection. Control regime titers were subtracted from mean end-point titers. Endpoint titer was defined as the x-axis intercept of the dilution curve at an absorbance value three standard deviations greater than the mean OD of pre-immune sera.

### Immunofluorescence Assay (IFA)

The ability of sera to recognize native Pfs25 on the surface of the *P. falciparum* ookinete/retort was assessed by IFA on bloodmeal extracts from parasite-fed *Anopheles gambiae* mosquitoes 26 hrs post-feed. *Briefly, P. falciparum* (NF54) gametocytes were cultured as described previously, and fed to pots of >50 *An. gambiae* mosquitoes^16^. 26 hrs post feed, fed mosquitoes were anesthetized, and engorged midguts containing semi-digested blood removed. These preparations were washed to remove excess blood, macerated, smeared onto glass slides, and were fixed in 4% PFA in PBS. IFA was performed as described in^17^. Control experiments from pre-immune mouse serum, and mice immunized with WT 2.34 ookinetes in each respective regime were performed.

### Direct Feeding Assay (DFA)

DFA was performed on immunized mice as described in^55^, with >50 *Anopheles stephensi* mosquitoes allowed to feed on each mouse. DFAs were performed in three tranches to account for varying experimental timings within regimes (Fig. 3A,B,C). Twelve days post-feed, midguts were dissected, and prevalence and intensity recorded. These values were compared to intensity and prevalence in non-immunized mice to assess inhibition of transmission.

### DMFA

To determine efficacy against field isolates of *P. falciparum*, children aged between 5–11 years in Bobo-Dioulasso, Burkina Faso, were screened for the presence of *P. falciparum* by thick blood smears. Gametocytemias ranging from 288-24 per µl blood were harvested to examine wide range of parasitic densities. 10 mL blood was drawn in heparinized tubes to obtain gametocytes. The plasma from the gametocyte positive donor blood was replaced by AB^+^ serum from a European donor, test (or control) serum was added at the stated dilution, and fed to *An. colluzi* mosquitoes. Mosquitoes were dissected day 7 post-feeding. Intensity, prevalence, and reduction in both were calculated as described previously. Mean observed control intensity/prevalence values were 48.3/80.8% (experiment 1), 65.1/96.1% (experiment 2), 1.2/50% (experiment 3).

### Ethical statement

All methods were carried out in accordance with relevant guidelines and regulations and were approved by the Imperial College Local Ethical Review Committee. Specifically, for animal studies, all procedures were performed in accordance with the UK Animals (Scientific Procedures) Act (UK Home Office License PPL 70/8788) and approved by Imperial College Animal Welfare and Ethical Review Body. For field/human studies, informed consent from all parents or guardians was obtained for children positive for gametocytes prior to blood sampling/DMFA (Protocol 003-2009/CE-CM, Centre Muraz institutional ethical committee).

### Data availability statement

The datasets generated during and/or analysed during the current study are available from the corresponding author on reasonable request.

### Statistical analysis

A regimen’s ability to reduce prevalence or intensity was assessed using generalized linear mixed effect models, GLMM^56^. GLMMs with and without antibody titre as a continuous linear effect were compared to investigate whether there was an association between titre and efficacy. Comparison between parametric ELISA tests were assessed using t-test. Statistical analysis was performed using R and Graphpad Prism. P values < 0.05 were considered statistically significant (*** =  < 0.001, ** = 0.001–0.01, * = 0.01–0.05).

### One Sentence Summary

A novel transgenic rodent malarial parasite induces potent transmission-blocking efficacy upon immunization.

## References

[1] WHO World Malaria report. World Health Organisation, http://www.who.int/malaria/publications/world_malaria_report/en/ (2017).

[2] Sinden RE (2010). A biologist’s perspective on malaria vaccine development. Hum. Vaccin.

[3] Rosenberg R (2008). Malaria: some considerations regarding parasite productivity. Trends Paras.

[4] The malERA Refresh Consultative Panel on Tools for Malaria Elimination. An updated research agenda for diagnostics, drugs, vaccines, and vector control in malaria elimination and eradication. *PLoS Med*. 10.1371/journal.pmed.1002455. 10.1371/journal.pmed.1002455 PMC5708606 29190291

[5] Kaslow DC, Bathurst IC, Barr PJ (1992). Malaria transmission-blocking vaccines. Trends Biotechnol..

[6] Niu G (2017). The fibrinogen-like domain of FREP1 protein is a broad-spectrum malaria transmission-blocking vaccine antigen. J Biol Chem..

[7] Vermeulen AN (1985). Sequential expression of antigens on sexual stages of *Plasmodium falciparum* accessible to transmission-blocking antibodies in the mosquito. J Exp Med.

[8] Kaslow DC (1988). A vaccine candidate from the sexual stage of human malaria that contains EGF-like domains. Nature.

[9] Lee SM (2016). Assessment of Pfs25 expressed from multiple soluble expression platforms for use as transmission-blocking vaccine candidates. Malaria Journal..

[10] Wu Y (2008). Phase 1 trial of malaria transmission-blocking vaccine candidates Pfs25 and Pvs25 formulated with montanide ISA 51. PLoS ONE.

[11] Talaat KR (2016). Safety and Immunogenicity of Pfs25-EPA/Alhydrogel, a Transmission Blocking Vaccine against *Plasmodium falciparum:* An Open Label Study in Malaria Naïve Adults. PLoS One.

[12] Zou L, Miles AP, Wang J, Stowers AW (2003). Expression of malaria transmission-blocking vaccine antigen Pfs25 in *Pichia pastoris* for use in human clinical trials. Vaccine..

[13] Kumar R, Angov E, Kumar N (2014). Potent malaria transmission blocking antibody responses elicited by *Plasmodium falciparum* Pfs25 expressed in *E.coli* after successful protein refolding. Infect Immun..

[14] Farrance CE (2011). Antibodies to plant-produced *Plasmodium falciparum* sexual stage protein Pfs25 exhibit transmission blocking activity. Hum Vaccin.

[15] Jones Chichester JA (2013). A Plant-Produced Pfs25 VLP Malaria Vaccine Candidate Induces Persistent Transmission Blocking Antibodies against *Plasmodium falciparum* in Immunized Mice. PLoS ONE..

[16] Goodman AL (2011). A viral vectored prime-boost immunization regime targeting the malaria Pfs25 antigen induces transmission-blocking activity. PLoS One...

[17] Kapulu MC (2015). Comparative assessment of transmission-blocking vaccine candidates against *Plasmodium falciparum*. Sci Rep..

[18] Blagborough AM, Yoshida S, Sattabongkot J, Tsuboi T, Sinden RE (2010). Intranasal and intramuscular immunization with Baculovirus Dual Expression System-based Pvs25 vaccine substantially blocks *Plasmodium vivax* transmission. Vaccine..

[19] Blagborough AM (2016). Transmission blocking potency and immunogenicity of a plant-produced Pvs25-based subunit vaccine against *Plasmodium vivax*. Vaccine..

[20] Wu Y (2006). Sustained high-titer antibody responses induced by conjugating a malarial vaccine candidate to outer-membrane protein complex. Proc Natl Acad Sci USA.

[21] Shimp RL (2013). Development of a Pfs25-EPA malaria transmission blocking vaccine as a chemically conjugated nanoparticle. Vaccine.

[22] Kumar R (2015). Nanovaccines for malaria using Plasmodium falciparum antigen Pfs25 attached gold nanoparticles. Vaccine.

[23] Li, Y. *et al*. Enhancing immunogenicity and transmission-blocking activity of malaria vaccines by fusing Pfs25 to IMX313 multimerization technology. *Sci Rep*. 18848 (2016). 10.1038/srep18848 PMC4705524 26743316

[24] Hoffman SL, Vekemans J, Richie TL, Duffy PE (2015). The March Toward Malaria Vaccines. Vaccine.

[25] Sauerwein RW, Richie TL (2015). Malaria vaccines getting close to clinical reality. Vaccine..

[26] Clyde DF, Most H, McCarthy VC, Vanderberg JP (1973). Immunization of man against sporozoite-induced falciparum malaria. Am J Med Sci.

[27] Rieckmann KH, Carson PE, Beaudoin RL, Cassells JS, Sell KW (1974). Sporozoite induced immunity in man against an Ethiopian strain of *Plasmodium falciparum*. Trans R Soc Trop Med Hyg.

[28] Gwadz RW (1976). Successful immunization against the sexual stages of *Plasmodium gallinaceum*. Science.

[29] Carter R, Chen DH (1976). Malaria transmission blocked by immunisation with gametes of the malaria parasite. Nature.

[30] Hoffman SL (2002). Protection of humans against malaria by immunization with radiation-attenuated *Plasmodium falciparum sporozoites*. J Infect Dis.

[31] Luke TC, Hoffman SL (2003). Rationale and plans for developing a non-replicating, metabolically active, radiation-attenuated *Plasmodium falciparum* sporozoite vaccine. J Exp Biol.

[32] Luke TC, Hoffman SL (2003). Rationale and plans for developing a non-replicating, metabolically active, radiation-attenuated *Plasmodium falciparum* sporozoite vaccine. J Exp Biol.

[33] Seder RA (2013). Protection against malaria by intravenous immunization with a nonreplicating sporozoite vaccine. Science.

[34] Roestenberg M (2009). Protection against a malaria challenge by sporozoite inoculation. N Engl J Med.

[35] Roestenberg M (2011). Long-term protection against malaria after experimental sporozoite inoculation: an open-label follow-up study. Lancet.

[36] Mordmüller B (2017). Sterile protection against human malaria by chemoattenuated PfSPZ vaccine. Nature..

[37] Sissoko MS (2017). Safety and efficacy of PfSPZ Vaccine against *Plasmodium falciparum* via direct venous inoculation in healthy malaria-exposed adults in Mali: a randomised, double-blind phase 1 trial. Lancet Infect Dispii.

[38] Lin JW (2011). A novel 'gene insertion/marker out' (GIMO) method for transgene expression and gene complementation in rodent malaria parasites. PLoS One.

[39] MVI-PATH portfolio; http://www.malariavaccine.org/projects/vaccine-projects/transgenic-p-berghei (2016).

[40] Good MF (2013). Cross-species malaria immunity induced by chemically attenuated parasites. J Clin Invest.

[41] Ting LM, Gissot M, Coppi A, Sinnis P, Kim K (2008). Attenuated *Plasmodium yoelii* lacking purine nucleoside phosphorylase confer protective immunity. Nat Med.

[42] McCarthy JS, Good MF (2010). Whole parasite blood stage malaria vaccines: a convergence of evidence. Hum Vaccin.

[43] Pinzon-Charry A (2010). Low doses of killed parasite in CpG elicit vigorous CD4+ T cell responses against blood-stage malaria in mice. J Clin Invest.

[44] Tomas AM (2001). P25 and P28 proteins of the malaria ookinete surface have multiple and partially redundant functions. EMBO J.

[45] Miura K (2007). Transmission-blocking activity induced by malaria vaccine candidates Pfs25/Pvs25 is a direct and predictable function of antibody titer. Malar J.

[46] Stanisic DI, Good MF (2015). Whole organism blood stage vaccines against malaria. Vaccine..

[47] Tsai CW, Duggan PF, Shimp RL, Miller LH, Narum DL (2006). Overproduction of *Pichia pastoris* or *Plasmodium falciparum* protein disulfide isomerase affects expression, folding and O-linked glycosylation of a malaria vaccine candidate expressed in *P. pastoris*. J Biotechnol..

[48] Carter V, Cable HC, Underhill AB, Jackie Williams J, Hurd H (2003). Isolation of *Plasmodium berghei* ookinetes in culture using Nycodenz density gradient columns and magnetic isolation. Malaria Journal.

[49] Ramakrishnan C (2013). Laboratory maintenance of rodent malaria parasites. Methods Mol Biol.

[50] Florens L (2002). A proteomic view of the *Plasmodium falciparum* life cycle. Nature. Oct.

[51] Hall N (2005). A comprehensive survey of the *Plasmodium* life cycle by genomic, transcriptomic, and proteomic analyses. Science..

[52] Lal K (2009). Characterisation of *Plasmodium* invasive organelles; an ookinete microneme proteome. Proteomics..

[53] Heidrich HG, Mrema JE, Vander Jagt DL, Reyes P, Rieckmann KH (1982). Isolation of intracellular parasites (*Plasmodium falciparum*) from culture using free-flow electrophoresis: separation of the free parasites according to stages. J Parasitol. Jun.

[54] Delves MJ (2012). A high-throughput assay for the identification of malarial transmission-blocking drugs and vaccines. Int J Parasitol. Oct.

[55] Blagborough AM (2013). Assessing transmission blockade in *Plasmodium spp*. Methods Mol Biol.

[56] Churcher TS (2012). Measuring the blockade of malaria transmission - An analysis of the Standard Membrane Feeding Assay. Int J Parasitol.

